# Castleman disease variant of POEMS syndrome without M protein: a case report

**DOI:** 10.3389/fonc.2024.1449945

**Published:** 2024-12-03

**Authors:** Min Ji, Shumin Jin, Shaolei Zang, Peng Li, Fei Lu, Chuanli Zhao, Chunqing Zhang, Chunyan Ji, Jingjing Ye

**Affiliations:** Department of Hematology, Qilu Hospital, Cheeloo College of Medicine, Shandong University, Jinan, China

**Keywords:** POEMS syndrome, Castleman disease, monoclonal protein, diagnostic criteria, case report

## Abstract

POEMS (polyneuropathy, organomegaly, endocrinopathy, monoclonal protein, skin changes) syndrome is a paraneoplastic syndrome associated with an underlying plasma cell neoplasm. According to the current diagnostic criteria for POEMS syndrome, the presence of characteristic polyneuropathy and clonal plasma cell disorder are required for diagnosis. We report a case of a Castleman disease variant of POEMS syndrome without monoclonal protein (M protein) expression, which presented with polyneuropathy, organomegaly, endocrinopathy, skin lesions, and sclerotic bone lesions. The patient was treated with lenalidomide and dexamethasone (RD), after which her symptoms improved. The findings in this case suggest that the diagnostic criteria for POEMS syndrome might require reconsideration.

## 1 Introduction

POEMS syndrome is a rare hematological disease characterized by polyneuropathy (P), organomegaly (O), endocrinopathy (E), monoclonal protein (M protein), and skin changes (S) that is prone to involve multiple organs and systems (1). Due to its clinical manifestations being diverse and non-specific, the clinical underdiagnosis and misdiagnosis rates are high, and patients would often have been running around multiple departments before a diagnosis is confirmed, with a median time between onset and diagnosis of about 18 months (2). It is generally considered that the diagnosis of POEMS syndrome requires the presence of polyneuropathy and monoclonal plasma cell disorder (3). In addition to the classic POEMS syndrome, there is a Castleman disease (CD) variant of this disease that might not be associated with polyneuropathy or M protein (4). We report a case of an M-protein-negative CD variant POEMS syndrome with the aim of improving the understanding of this disease and reducing the misdiagnosis and underdiagnosis rates.

## 2 Case presentation

A 54-year-old Chinese woman was admitted to Qilu Hospital with symptoms of weakness and numbness of both lower limbs for 3 months, as well as abdominal distension and edema of both lower limbs for 1 month, with no fever or night sweats. She once came to the local hospital, where her laboratory investigations showed hypothyroidism, the anti-nuclear antibody was weakly positive, and the anti-SSA antibody was positive. CT scan of the chest and abdomen indicated enlarged lymph nodes in the neck, supraclavicular and bilateral axillae, and mediastinum; bilateral pleural effusion, ascites, and splenomegaly; bilateral pleural thickening; and pulmonary hypertension. Gastroscopy showed extensive redness, edema, and erosion of the mucosa in the fundus and body of the stomach. Colonoscopy did not show any abnormality. The patient was treated with a diuretic, euthyrox substitute treatment, but the effect was poor. The patient had gained 5 kg in weight since the onset of the disease. The patient had a history of hypertension for more than 10 years, was treated with oral valsartan, and was allergic to penicillin.

**Figure 1:**
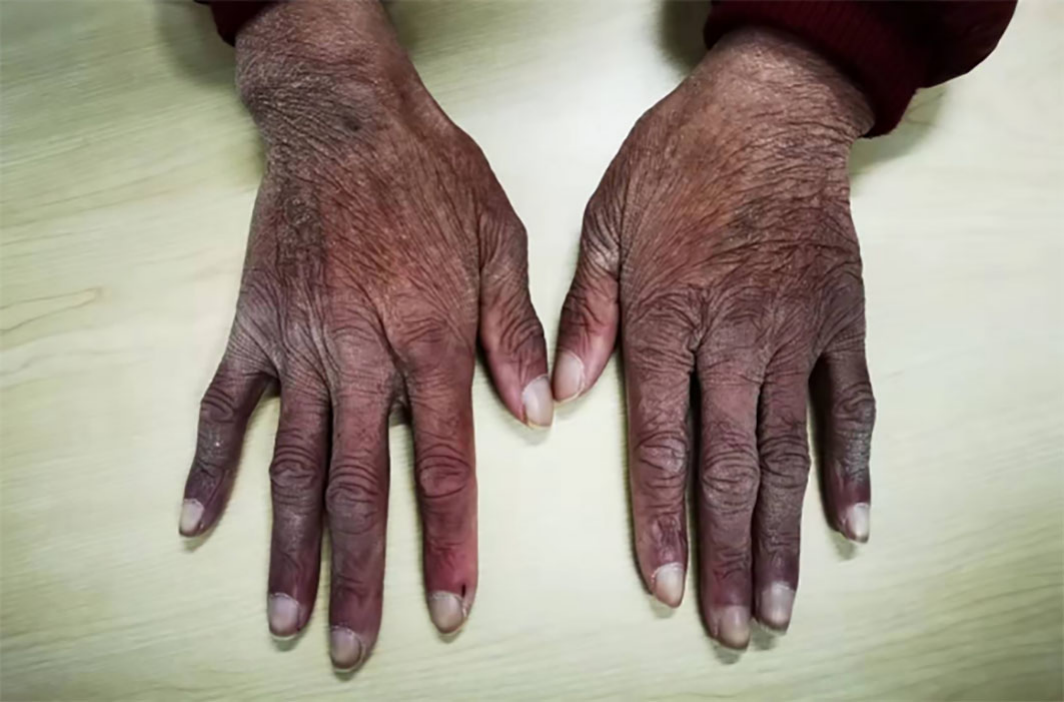
Pigmentation in the patient’s skin and white fingernails.

On admission, pigmentation was noted on the patient’s skin, and the patient had facial atrophy (**Figure 1**). Enlarged lymph nodes were palpable on both sides of the neck, supraclavicular fossa, axilla, and groin, which were approximately 2 cm × 3 cm in size, tough in texture, and without pressure pain. The abdomen was distended, with shifting dullness (+). Her lower extremities showed moderately depressed edema. The myodynamia of both upper limbs was grade 4, while that of both lower limbs was grade 2. The patient presented with sock-like hyperalgesia in the extremities and with decreased tendon reflexes in both lower extremities.

After admission, the patient underwent relevant auxiliary examination. The erythrocyte sedimentation rate (ESR) level was 60 mm/h, while the C-reactive protein was 15.5 mg/L. The patient’s thyroid function and liver function were decreased, with thyroid-stimulating hormone, free triiodothyronine, free thyroxine, and serum albumin levels of 5.38 μIU/L (normal range = 0.270–4.200 μIU/L), 1.45 pmol/L (normal range = 3.10–6.80 pmol/L), 9.98 pmol/L (normal range = 12.00–22.00 pmol/L), and 29.9 g/L (normal range = 40.0–55.0 g/L), respectively. Her prolactin level was 1,097 μIU/ml (normal range = 102.00–496.00 μIU/ml), and rheumatic series revealed anti-nuclear antibody 1:100 (+) and anti-SSA antibody (+). Moreover, for anti-cardiolipin antibodies (ACAs), immunoglobulin A (IgA) was 5.4 RU/ml (normal range = 0–4.2 RU/ml) and IgM was 7.3 RU/ml (normal range = 0–14.5 RU/ml). There were no obvious abnormalities in lupus anticoagulant. The serological HIV test was negative.

The patient underwent serum protein electrophoresis and immunofixation electrophoresis, and no M protein was detected. The patient’s serum IgG, Igκ light chain, and Igλ light chain levels were 14.2 g/ml (normal range = 7.00–16.00 g/ml), 3.23 g/L (normal range = 1.70–3.70 g/L), and 2.87 g/L (normal range = 0.9–2.1 g/L), respectively, while the urinary Igκ and Igλ light chain levels were 161 mg/L (normal range < 7.19 mg/L) and 143 mg/L (normal range < 4.10 mg/L), respectively. The urinary Igκ/Igλ free light chains were normal. There was no M protein expression detected in the serum, in urine immunofixation, and in urine electrophoresis. Her serum vascular endothelial growth factor (VEGF) level was 322.43 pg/ml (normal range = 0–142 pg/ml).

**Figure 2:**
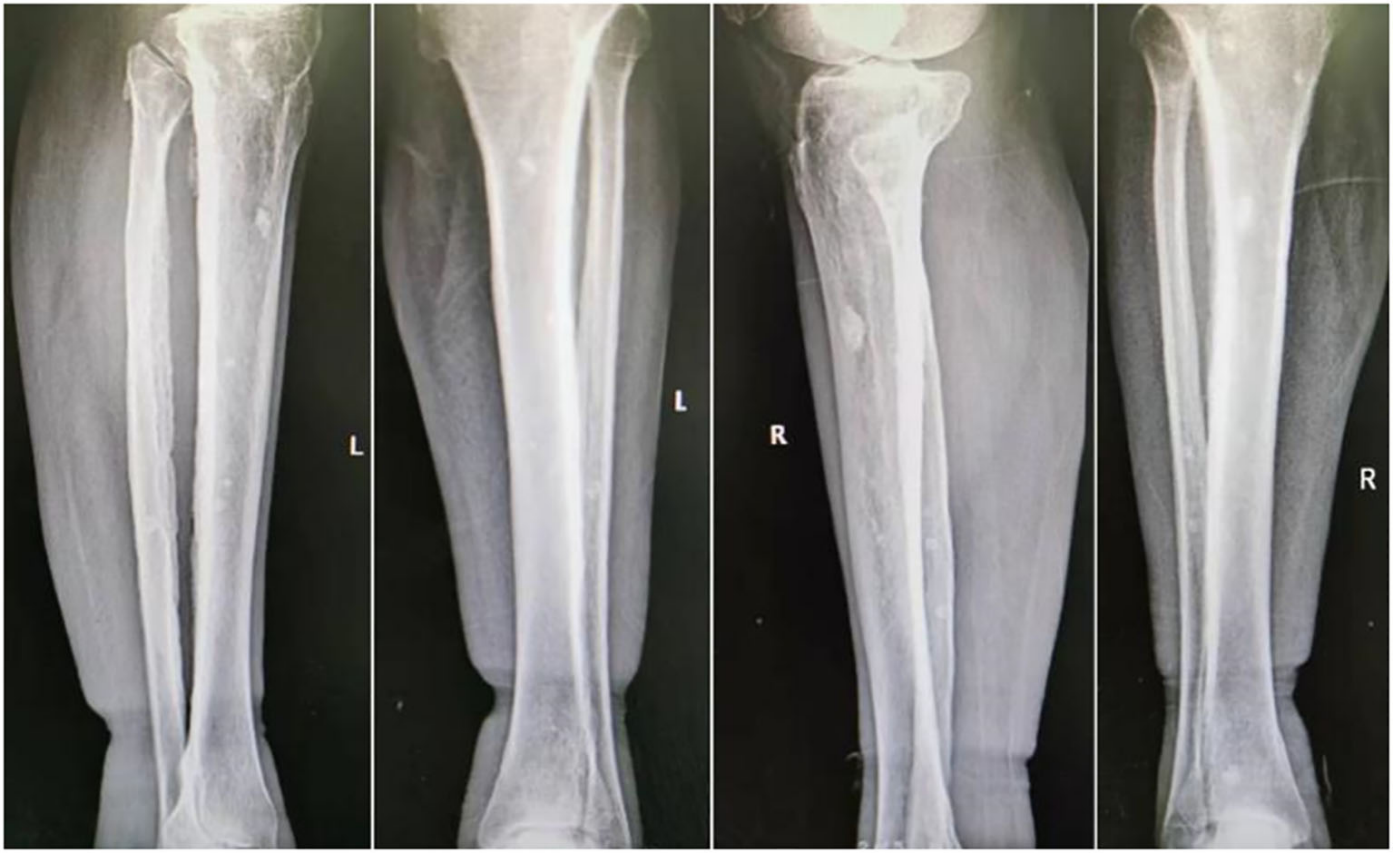
Osteosclerotic lesions in the patient’s legs.

Ascitic fluid puncture tests showed that the ascites was exudative, with no bacteria, fungi, or acid-fast bacilli being detected. The ultrasonic cardiogram showed an enlarged left atrium, slightly widened pulmonary arteries, moderate pulmonary hypertension, and a small amount of pericardial effusion. X-ray showed the bilateral tibiofibular bone to be cortically hyperplastic and gross, with multiple patchy, hyperdense shadows (**Figure 2**). Fundus examination showed papillary edema of the optic nerve. **Tables 1**, **2** present the patient’s electromyogram and the motor nerve and sensory nerve conduction velocity measurements. It can be seen that the patient had severe peripheral neuropathy of the upper and lower extremities with cumulative motor and sensory nerves. The skin sympathetic reactions of both lower extremities were abnormal.

**Table 1:** Motor nerve conduction test.

Motor Nerve		Latency		Amplitude				Conduction velocity
		M distal latency	Negative wave duration difference	Negative amplitude	Negative wave amplitude difference	Negative wave area		
		ms	%	mV	%	ms*mV	ms	m/s
left ulnar nerve	Wrist-ADM	3.75↑		6.5 (-)		27.3	7.3	
	BelElb-Wrist	11.3	9.6	5.4	-16.9	26.2	8	30.5↓↓
left median nerve	Wrist-APM	4.58↑		4.3 (-)		20.5	9.1	
	BelElb-Wrist	11.2	2.2	2.5	-41.9	11.8	9.3	34.0↓↓
left Tibialls	Ankle-AH	––		––		––	––	
	Popliteus - Gastrocnemius muscle	7.88↑↑		0.050↓↓↓		0.3	11.3	
	Knee-Ankle	37.3	––	0.034↓↓↓	––	––	––	
right Tibialls	Ankle-AH	––		––		––	––	
	Popliteus - Gastrocnemius muscle	––		––		––	––	
	Knee-Ankle	––	––	––	––	––	––	
Left common peroneal nerve tibialis anterior muscle	Caput fibulae-Tib.ant	4.99↑		0.17↓↓↓		1.04	10.3	
right common peroneal nerve tibialis anterior muscle	Caput fibulae-Tib.ant	4.81↑		0.087↓↓↓		0.35	8.5	
Left deep peroneal nerve	Ankle-EDB	––		––		––	––	
	Caput fibulae-Ankle							––
right deep peroneal nerve	Ankle-EDB	––		––		––	––	
	Caput fibulae-Ankle		––		––			––

The symbols “↑” and “↑↑” refer to lightly and moderately prolonged, respectively. The symbols “↓↓” and “↓↓↓” refer to a moderate and severe reduction. The symblos “––” means not drawn out. The symbol “(-)” stands for negative.

**Table 2:** Sensory nerve conduction test.

Nerve	Stimulation site	Latent period	Amplitude	Conduction velocity
left ulnar nerve	Finger V - wrist	3.09	12.2 (-)	42.1↓
left median nerve	Finger II - wrist	3.8	12.8 (-)	39.5↓

The symbol “↓” refers to a light decrease. The symbol “(-)” stands for negative.

**Figure 3:**
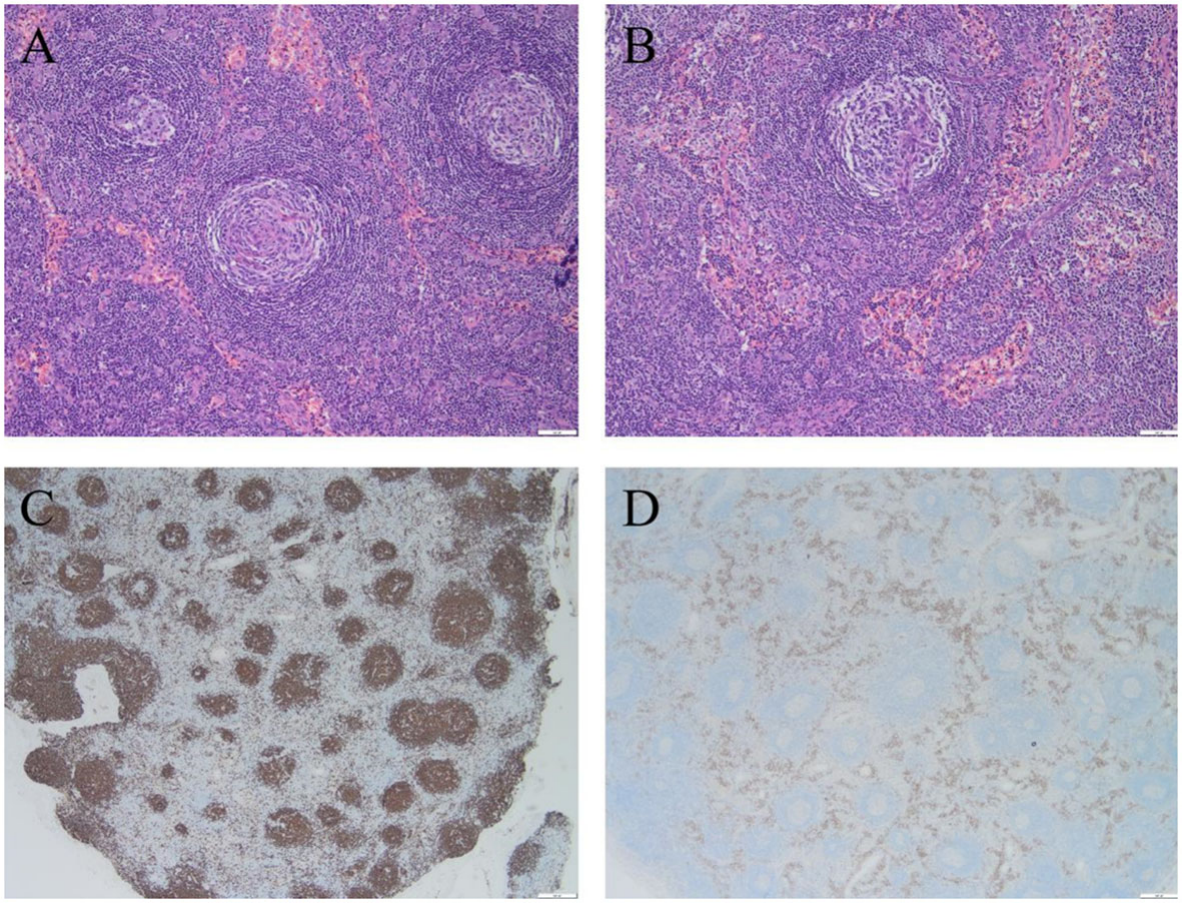
Biopsy pathology of the patient's axillary lymph node revealed Castleman disease, hyaline vascular type. **(A)** Hyaline vascular Castleman disease target ring arrangement, H & E stainig, ×100; **(B)** Hyaline vascular Castleman disease “lollipop”-like structures, H & E stainig, ×100; **(C)** CD20 stainig, ×20; **(D)** CD38 staining, ×20.

Bone marrow aspiration revealed occasional mature plasma cells seen at the caudal end of the slice, while biopsy revealed that a few plasma cells were scattered. Immunophenotyping by flow cytometry showed total plasma cells occupying 2.07% of the nucleated cells, but no phenotypic abnormalities were observed. It was suggested that the patient had no evidence of monoclonality of the plasmacytoid cells. The axillary lymph node biopsy pathology showed CD of the hyaline vascular type (**Figure 3**). Immunohistochemistry showed the following: B cells: CD20(+) and CD79a(+); CD3 (T cells+); CD21 (FDC+); plasma cells: CD38(+), CD138(+), MUM-1(+), Kappa(+), Lambda(+), IgG(+), and IgG-4(individual+); germinal centers: CD10(+), Bcl-6(+), cyclin D1(−), IgD(set area+), Bcl-2(+), TDT(−), HHV8(−), and a Ki-67 positivity rate of approximately 20%; *in situ* hybridization: EBER(−).

According to the diagnostic criteria for POEMS syndrome proposed by the Mayo Clinic in 2007 (5), two mandatory criteria, at least one major criterion, and at least one minor criterion are needed for diagnosis. The mandatory criteria are as follows: 1) polyneuropathy and 2) monoclonal plasma cell proliferative disorder (M protein positivity or plasmacytoma). The primary criteria are as follows: 1) Castleman disease; 2) sclerotic bone lesions; and 3) elevated serum or plasma VEGF levels. The secondary criteria are as follows: 1) organ enlargement; 2) increased water load; 3) endocrinopathy; 4) skin changes; 5) optic papilledema; and 6) thrombocytosis/erythrocytosis. The patient had typical multiple peripheral neuropathy, high serum VEGF, and CD, as well as extravascular volume overload, organomegaly, endocrinopathy, and typical skin changes. However, the patient lacked evidence of monoclonal plasma cell abnormalities. In the diagnostic update for POEMS syndrome published in the American Journal of Hematology in 2011 (6), a Castleman lesion atypia of POEMS syndrome, which can present without evidence of clonal plasma cell abnormalities, was mentioned. After a multidisciplinary discussion, the patient was diagnosed with a CD variant of POEMS syndrome without M protein based on one mandatory criterion, three major criteria, and five minor criteria.

As the patient did not have a desire for autologous stem cell transplantation (ASCT), she was treated with the lenalidomide and dexamethasone (RD) combined regimen (lenalidomide 25 mg/day on days 1-21 and dexamethasone 20 mg/day on days 1, 2, 8, 9, 15, 16, 22, and 23, in a course of 28 days). At the same time, a diuretic, albumin supplementation, triple cephalosporin anti-infection, and thyroxine replacement therapy were given.

After three cycles of treatment, there was less skin pigmentation than before and the nerve symptoms and edema of both lower extremities improved. The patient felt that the weakness in both lower limbs was reduced and that the limbs were stronger than before. Laboratory tests revealed a prolactin level of 1,590 μIU/ml and a serum VEGF value of 878.78 pg/ml, suggesting poor prognosis for the patient.

## 3 Discussion

POEMS syndrome is a rare plasma cell disorder with an incidence of less than one in 1 million. Its pathogenesis is not well understood. Due to its rarity, multisystem involvement, and high clinical heterogeneity, it has high rates of underdiagnosis and misdiagnosis. The most common initial symptoms of POEMS syndrome are peripheral neuropathy and increased water burden (pleural effusion, edema, and ascites), with the first departments involved being mostly neurology, nephrology, or gastroenterology, which can be easily misdiagnosed as chronic inflammatory demyelinating polyradiculoneuropathy (CIDP), chronic nephritis, or tuberculous peritonitis (7). In this case, the first symptom was numbness of both lower extremities, and the patient gradually developed abdominal distension and edema, which were not clearly diagnosed in the local hospital. As the patient also had skin pigmentation, hypothyroidism, lymph node enlargement, and splenomegaly, the gastroenterology department highly suspected POEMS syndrome and asked the hematology department for assistance, at the same time made a differential diagnosis of diseases causing ascites, including tumor, tuberculosis, cirrhosis, and connective tissue disease. Ultimately, the patient was able to get a definitive diagnosis and a timely treatment.

The diagnosis of POEMS syndrome requires the fulfillment of two mandatory criteria, at least one major criterion, and at least one minor criterion. Cases that meet these criteria have classic POEMS syndrome. In addition, there are a few atypical POEMS syndromes. Suichi et al. surveyed 392 cases of POEMS syndrome and found that only 89% had monoclonal plasma cell proliferative disorder (8). He et al. reported 13 cases of the CD variant POEMS syndrome without M protein. All of these patients had typical neuropathy, CD, sclerotic bone lesions, and VEGF >2,000 ng/L, all secondary criteria. The patients were treated with anti-plasmapheresis, and all had good outcomes (9). According to previous studies, negative M protein assays have been attributed to the following. Firstly, limited levels of detection: serum protein electrophoresis (sPEP), immunofixation electrophoresis (sIFE), and serum free light chain (sFLC), as the main methods for the detection of M protein, have improved the diagnosis of malignant plasma cell disease; however, in patients with POEMS syndrome, there are still cases of negative M protein assays (10). Secondly, the M protein levels are below the detection threshold and may be in the “prophase” of the disease or in clinical remission after systemic therapy (11). Thirdly, non-secretory plasma cells may be present in POEMS syndrome. In addition, there is also a variant of POEMS syndrome without polyneuropathy (2). Morizane et al. reported on a 43-year-old Japanese woman with organomegaly, endocrinopathy, M protein, skin lesions, and typical renal and sclerotic bone lesions. However, the neurological examinations and peripheral nerve conduction tests were both normal 5 years since her skin lesions appeared (12). In fact, the polyneuropathy in these patients is only on the way, and as the disease progresses, the patient will eventually develop peripheral neuropathy and become classic POEMS syndrome (7). However, it is important to note that the diagnosis of variant POEMS syndrome should not be expanded blindly, and in the absence of M protein or polyneuropathy, almost all other diagnostic criteria need to be met for the diagnosis to be made.

Castleman disease (CD), also known as giant lymphadenopathy or vascular follicular lymphadenopathy, is included in the first list of rare diseases. It is pathologically classified into the hyaline-vascular, plasma cell, and mixed types (13) and clinically into unicentric CD (UCD) and multicentric CD (MCD) (14). MCD is subdivided into human herpes virus type 8 (HHV-8)-associated MCD and idiopathic multicentric CD (iMCD) on the basis of the HHV-8 and human immunodeficiency virus (HIV) infection status. In addition to enlarged lymph nodes, MCD is often associated with fever, night sweats, malaise, weight loss, anemia, hypohepatia, renal insufficiency, excessive volume load (systemic edema, pleural fluid, ascites, etc.), and other manifestations. As many diseases (including malignant tumors, infectious diseases, and autoimmune diseases) are also associated with “Castleman-like” pathological changes in the lymph nodes, the first step in the diagnosis of CD is to exclude related diseases that may be associated with similar pathological changes in the lymph nodes of CD, including POEMS syndrome, tuberculosis, and systemic lupus erythematosus, among others (13). Pathological biopsy of the diseased tissue can assist the diagnosis of CD (**Figure 3**). However, in patients who can be diagnosed with CD but are fully compatible with the diagnosis of POEMS syndrome, CD should be used as one of the main diagnostic criteria for POEMS syndrome rather than as an individual diagnosis (15). For some UCD patients, when possible, complete surgical removal of the lesion is preferred. UCD has a positive prognosis, with a 5-year survival rate of more than 90%. For HHV-8-positive MCD, rituximab-based therapy is available.

POEMS syndrome is a chronic course disease, and the overall survival (OS) rate of a typical POEMS is 79% according to the Mayo study (16). In a long-term follow-up for M-protein-negative POEMS syndrome, the median progression-free survival (PFS) was 101.5 months (9). In 2017, Wang et al. developed a stratified prognostic model for POEMS syndrome. The 10-year OS rates were 98%, 75%, and 50% for the low-, medium-, and high-risk groups, respectively. According to this prognostic stratification model, the patient’s prognosis was stratified as high risk (17).

As few randomized clinical trials have been published for patients with POEMS syndrome, there have been no standard treatment for this disease (18). Radiation therapy is recommended for patients with isolated bone lesions (19, 20). Once disseminated disease is detected, anti-plasma cell therapy is the mainstay of treatment, including ASCT, melphalan, lenalidomide, thalidomide, and bortezomib combined with dexamethasone-based therapies (21–24). Studies have confirmed that ASCT is effective in POEMS syndrome, which was first published in 2002 (25). Almost 100% of patients had obvious clinical improvement, and the survival rates were very high (26–28). Melphalan is one of the most effective drugs for the treatment of plasma cell disease. In a single-center prospective study of 31 patients who received 12 cycles of melphalan and dexamethasone, 80.6% had a hematologic response, and all had neurologic improvement (21). In addition, thalidomide and lenalidomide, which have anti-VEGF and anti-TNF effects, have been used in the treatment of POEMS syndrome. Li et al. conducted a 41-cycle phase II trial of lenalidomide and dexamethasone in 12 patients, which showed a neurological response rate of 95%, complete hematologic response rate of 46%, and a VEGF response rate of 83% (29). Nozza et al. also reported the results of a prospective study of 18 patients treated with lenalidomide and dexamethasone (30). In addition, a study reported on more than 30 patients who were treated with bortezomib (31), while another retrospective study treated 20 patients with a reduced dose, all with good results (32). VEGF is a specific diagnostic indicator of POEMS syndrome, which can indicate the recurrence and progression of the disease (33). Bevacizumab is a monoclonal antibody against VEGF, which can be used in POEMS in theory. However, its clinical effect is still unclear (34).

As the patient had no intention of undergoing hematopoietic stem cell transplantation, she was treated with the RD regimen. Reexamination after three cycles of treatment showed that the skin pigmentation of the patient was less than before and that the muscle strength of both lower limbs was grade 4, suggesting that the treatment was effective.

## 4 Conclusion

In conclusion, we report a case of POEMS syndrome without M protein expression, in which the patient presented with polyneuropathy, multiple plasmacytoid effusions, skin pigmentation, white nails, CD, an elevated blood VEGF, sclerosing bone disease, elevated prolactin, hypothyroidism, optic papillae edema, and pulmonary hypertension. POEMS syndrome is a rare disease with complex clinical manifestations involving the nervous system, the endocrine system, the hematological system, and other areas, with an insidious onset and non-specific initial symptoms, making it easy to be misdiagnosed and underdiagnosed. This case suggests that clinicians ought to expand clinical thinking and to recognize and identify characteristic changes in POEMS syndrome in order to reduce the underdiagnosis rate of this disease. Furthermore, the existing nomenclature and diagnostic criteria for POEMS syndrome might need to be reassessed.

## Data Availability

The original contributions presented in the study are included in the article/supplementary material. Further inquiries can be directed to the corresponding author.

## References

[1] Ali T Qazilbash MH. POEMS syndrome: A multisystem clonal disorder. Eur J Haematol. (2021) 106:14–8. doi: 10.1111/ejh.13514 32889731

[2] Li J Zhou DB Huang Z Jiao L Duan MH Zhang W. Clinical characteristics and long-term outcome of patients with POEMS syndrome in China. Ann Hematol. (2011) 90:819–26. doi: 10.1007/s00277-010-1149-0 21221584

[3] Dispenzieri A Kyle RA Lacy MQ Rajkumar SV Therneau TM Larson DR. POEMS syndrome: definitions and long-term outcome. Blood. (2003) 101:2496–506. doi: 10.1182/blood-2002-07-2299 12456500

[4] Dispenzieri A. POEMS syndrome: 2021 Update on diagnosis, risk-stratification, and management. Am J Hematol. (2021) 96:872–88. doi: 10.1002/ajh.26240 34000085

[5] Dispenzieri A. POEMS syndrome. Blood Rev. (2007) 21:285–99. doi: 10.1016/j.blre.2007.07.004 17850941

[6] Dispenzieri A. POEMS syndrome: 2011 update on diagnosis, risk-stratification, and management. Am J Hematol. (2011) 86:591–601. doi: 10.1002/ajh.22050 21681783

[7] Mauermann ML Sorenson EJ Dispenzieri A Mandrekar J Suarez GA Dyck PJ. Uniform demyelination and more severe axonal loss distinguish POEMS syndrome from CIDP. J Neurol Neurosurg Psychiatry. (2012) 83:480–6. doi: 10.1136/jnnp-2011-301472 22396441

[8] Suichi T Misawa S Beppu M Takahashi S Sekiguchi Y Shibuya K. Prevalence, clinical profiles, and prognosis of POEMS syndrome in Japanese nationwide survey. Neurology. (2019) 93:e975–83. doi: 10.1212/wnl.0000000000008062 31371568

[9] He T Zhao A Zhao H Cai H Feng J Zhang L. Clinical characteristics and the long-term outcome of patients with atypical POEMS syndrome variant with undetectable monoclonal gammopathy. Ann Hematology. (2019) 98:735–43. doi: 10.1007/s00277-018-03589-4 30612232

[10] Sethi S Theis JD Leung N Dispenzieri A Nasr SH Fidler ME. Mass spectrometry-based proteomic diagnosis of renal immunoglobulin heavy chain amyloidosis. Clin J Am Soc Nephrol. (2010) 5:2180–7. doi: 10.2215/CJN.02890310 PMC2994078 20876678

[11] Wang C Su W Cai QQ Cai H Ji W Di Q. Prognostic value of serum heavy/light chain ratios in patients with POEMS syndrome. Eur J Haematol. (2016) 97:48–54. doi: 10.1111/ejh.12682 26383741

[12] Morizane R Sasamura H Minakuchi H Takae Y Kikuchi H Yoshiya N. A case of atypical POEMS syndrome without polyneuropathy. Eur J Haematol. (2008) 80:452–5. doi: 10.1111/j.1600-0609.2008.01045.x 18284621

[13] Fajgenbaum DC Uldrick TS Bagg A Frank D Wu D Srkalovic G. International, evidence-based consensus diagnostic criteria for HHV-8–negative/idiopathic multicentric Castleman disease. Blood. (2017) 129:1646–57. doi: 10.1182/blood-2016-10-746933 PMC5364342 28087540

[14] Carbone A Borok M Damania B Gloghini A Polizzotto MN Jayanthan RK. Castleman disease. Nat Rev Dis Primers. (2021) 7:84. doi: 10.1038/s41572-021-00317-7 34824298 PMC9584164

[15] Huishou Fan WY Liu J Du C Xu Y Deng S Sui W. Analysis of misdiagnosis and missed diagnosis of POEMS with respect to hospital visit patterns. Chin J Of Clin Oncol. (2021) 48:1120–4. doi: 10.12354/j.issn.1000-8179.2021.20210665

[16] Dispenzieri A. POEMS syndrome: 2017 Update on diagnosis, risk stratification, and management. Am J Hematol. (2017) 92:814–29. doi: 10.1002/ajh.24802 28699668

[17] Wang C Huang XF Cai QQ Cao XX Duan MH Cai H. Prognostic study for overall survival in patients with newly diagnosed POEMS syndrome. Leukemia. (2017) 31:100–6. doi: 10.1038/leu.2016.168 27338259

[18] Kuwabara S DA Arimura K Misawa S Nakaseko C. Treatment for POEMS (polyneuropathy, organomegaly, endocrinopathy, M-protein, and skin changes) syndrome. Cochrane Database Syst Rev. (2012) 6):CD006828. doi: 10.1002/14651858.CD006828.pub3 PMC7389818 22696361

[19] Humeniuk MS Gertz MA Lacy MQ Kyle RA Witzig TE Kumar SK. Outcomes of patients with POEMS syndrome treated initially with radiation. Blood. (2013) 122:68–73. doi: 10.1182/blood-2013-03-487025 23699599 PMC4067496

[20] Suh YG Suh CO Kim JS Kim SJ Pyun HO Cho J. Radiotherapy for solitary plasmacytoma of bone and soft tissue: outcomes and prognostic factors. Ann Hematol. (2012) 91:1785–93. doi: 10.1007/s00277-012-1510-6 22752147

[21] Li J Zhang W Jiao L Duan MH Guan HZ Zhu WG. Combination of melphalan and dexamethasone for patients with newly diagnosed POEMS syndrome. Blood. (2011) 117:6445–9. doi: 10.1182/blood-2010-12-328112 PMC3123016 21393478

[22] Zhao H X-f H X-m G Cai H Zhang L Feng J. What is the best first-line treatment for POEMS syndrome: autologous transplantation, melphalan and dexamethasone, or lenalidomide and dexamethasone? Leukemia. (2019) 33:1023–9. doi: 10.1038/s41375-019-0391-2 PMC6756085 30700844

[23] Misawa S Sato Y Katayama K Nagashima K Aoyagi R Sekiguchi Y. Safety and efficacy of thalidomide in patients with POEMS syndrome: a multicentre, randomised, double-blind, placebo-controlled trial. Lancet Neurol. (2016) 15:1129–37. doi: 10.1016/S1474-4422(16)30157-0 27496680

[24] Dispenzieri A Lacy MQ Hayman SR Kumar SK Buadi F Dingli D. Peripheral blood stem cell transplant for POEMS syndrome is associated with high rates of engraftment syndrome. Eur J Haematol. (2008) 80:397–406. doi: 10.1111/j.1600-0609.2008.01037.x 18221391 PMC2327207

[25] Jaccard A Royer B Bordessoule D Brouet JC Fermand JP. High-dose therapy and autologous blood stem cell transplantation in POEMS syndrome. Blood. (2002) 99(8):3057–9. doi: 10.1182/blood.v99.8.3057 11929800

[26] Dispenzieri A Moreno-Aspitia A Suarez GA Lacy MQ Colon-Otero G Tefferi A. Peripheral blood stem cell transplantation in 16 patients with POEMS syndrome, and a review of the literature. Blood. (2004) 104:3400–7. doi: 10.1182/blood-2004-05-2046 15280195

[27] D'Souza A Lacy M Gertz M Kumar S Buadi F Hayman S. Long-term outcomes after autologous stem cell transplantation for patients with POEMS syndrome (osteosclerotic myeloma): a single-center experience. Blood. (2012) 120:56–62. doi: 10.1182/blood-2012-04-423178 22611150

[28] Cook G Iacobelli S van Biezen A Ziagkos D LeBlond V Abraham J. High-dose therapy and autologous stem cell transplantation in patients with POEMS syndrome: a retrospective study of the Plasma Cell Disorder sub-committee of the Chronic Malignancy Working Party of the European Society for Blood & Marrow Transplantation. Haematologica. (2017) 102:160–7. doi: 10.3324/haematol.2016.148460 PMC5210246 27634201

[29] Li J Huang XF Cai QQ Wang C Cai H Zhao H. A prospective phase II study of low dose lenalidomide plus dexamethasone in patients with newly diagnosed polyneuropathy, organomegaly, endocrinopathy, monoclonal gammopathy, and skin changes (POEMS) syndrome. Am J Hematol. (2018) 93:803–9. doi: 10.1002/ajh.25100 29603764

[30] Nozza A Terenghi F Gallia F Adami F Briani C Merlini G. Lenalidomide and dexamethasone in patients with POEMS syndrome: results of a prospective, open-label trial. Br J haematology. (2017) 179:748–55. doi: 10.1111/bjh.14966 29048107

[31] Zeng K Yang JR Li J Wei Q Yang YM Liu T. Effective induction therapy with subcutaneous administration of bortezomib for newly diagnosed POEMS syndrome: a case report and a review of the literature. Acta haematologica. (2013) 129:101–5. doi: 10.1159/000343681 23171959

[32] He H Fu W Du J Jiang H Hou J. Successful treatment of newly diagnosed POEMS syndrome with reduced-dose bortezomib based regimen. Br J haematology. (2018) 181:126–8. doi: 10.1111/bjh.14497 28146276

[33] Cao X Wang C Cai H Duan M Zhang W Li T. Diagnostic performance and clinical correlation of serum vascular endothelial growth factor levels in patients with newly diagnosed POEMS syndrome. Zhonghua Xue Ye Xue Za Zhi. (2014) 35(12):1065–8. doi: 10.3760/cma.j.issn.0253-2727.2014.12.004 25543698

[34] Brown R Ginsberg L. POEMS syndrome: clinical update. J neurology. (2019) 266:268–77. doi: 10.1007/s00415-018-9110-6 PMC6342878 30498913

